# PERK/ATF3-Reduced ER Stress on high potassium environment in the suppression of tumor ferroptosis

**DOI:** 10.7150/jca.83556

**Published:** 2023-05-15

**Authors:** Xufeng Pu, Li Li, Zhenhui Chen, Aihua Gong, Jiao Lei, Lirong Zhang, Hsiang-I Tsai

**Affiliations:** 1 Institute of Medical Imaging and Artificial Intelligence, 212001, Zhenjiang, Jiang Su Province, China.; 2 Department of Medical Imaging, The Affiliated Hospital of Jiangsu University, 212001, Zhenjiang, China.; 3 Department of Cell Biology, School of Medicine, Jiangsu University, 212001, Zhenjiang, Jiang Su Province, China.; 4 Nanjing Lishui People's Hospital, Zhongda Hospital Lishui Branch, Southeast University, Nanjing, Jiang Su Province, China.

## Abstract

Potassium (K^+^) is a vital intracellular cation. In the human body, it regulates membrane potential, electrical excitation, protein synthesis, and cell death. Recent studies revealed that dying cancer cells release potassium into the tumor microenvironment (TME), thereby influencing cell survival-related events. Several investigations reported that potassium channels and high potassium levels influence apoptosis. Increasing extracellular potassium and inhibiting K^+^ efflux channels significantly block the apoptotic machinery. However, it is unknown whether a high-potassium environment also affects other types of cell death such as ferroptosis. In the present study, cell counting kit (CCK-8), colony formation ability, and 5-ethynyl-2′-deoxyuridine (EdU) assays demonstrated that a high-potassium environment reverses erastin-induced ferroptosis. RNA sequencing (RNA-Seq) and Kyoto Encyclopedia of Genes and Genomes (KEGG) and gene ontology (GO) analyses indicated that high potassium levels attenuated the unfolded protein response that is characteristic of endoplasmic reticulum (ER) stress. The ER transmembrane proteins PRKR-like ER kinase (PERK), inositol-requiring enzyme 1α (IRE1α), and activating transcription factor 6 (ATF6) are recognized as ER stress sensors. Here, the PERK blocker GSK2606414 significantly rescued ferroptosis. The present work also disclosed that the ER-related gene activating transcription factor 3 (ATF3*)* played a vital role in regulating ferroptosis in a high-potassium environment. The foregoing results revealed the roles of potassium and the TME in cancer cell ferroptosis and provided a potential clinical therapeutic strategy for cancer.

## Introduction

Homeostasis of metal ions such as copper, iron, zinc, and calcium are crucial for the cell survival, function and normal physiological activity of the brain. However, tumorigenesis upsets metal cation balance 1. Previous research demonstrated that the concentrations of Fe, K, Mg, Na, Rb, Se, and Zn were significantly higher in neoplastic than in normal tissues 2, 3. Recent studies revealed that the potassium ion content increases mainly in the extracellular milieu in response to cell death. High extracellular potassium levels favor cell survival by suppressing T-cell function 4. Nevertheless, the mechanism by which a high-potassium environment influences cancer cells is unknown. A few studies indicated that high extracellular potassium levels inhibit apoptosis. Nevertheless, the effects of elevated extracellular K^+^ on other types of cell death have not yet been determined 5.

Unlike other forms of apoptosis, ferroptosis is driven by iron-dependent phospholipid peroxidation and oxidative stress 6 and is regulated by mitochondrial activity, redox homeostasis, and amino acid metabolism 7. Ferroptosis is associated with high oxidative stress and is, therefore, a novel therapeutic target in refractory tumors. For example, as ferroptosis induction targets *GPX4^8^,* it might be a therapeutic strategy for the luminal androgen receptor subtype of triple-negative breast cancers. Several authors reported that the TME influences ferroptosis-inducing pathways such as hypoxia, lactate, and low glucose in cancer cells 9-11. Nevertheless, it remains to be determined how a high-potassium environment affects ferroptosis.

In the present study, we confirmed that a high-potassium environment protects cells against ferroptosis. RNA sequencing disclosed that high potassium levels alleviated ER stress associated with ferroptosis induction. We also discovered that inhibiting the ER-related gene *ATF3* played a vital role in a ferroptosis-inducted high-potassium environment.

## Methods

### Reagents

(1S,3R)-RSL3 (RSL3, #HY-100218A), erastin (#HY-15763), ferrostatin-1 (#HY-100579), Z-VAD-FMK (#HY-16658B), necrostastin-1 (#HY-15760), necrostatin-2 (#HY-14622), GSK2606414 (#HY-18072), and piperazine erastin (#HY-100887) were purchased from MedChemExpress (MCE; Monmouth Junction, NJ, USA). Ceapin-A7 (T9110) and 4μ8C (T6363) were purchased from MedChemExpress.

### Cell culture and stable cell line establishment

HT1080, HepG2, and HEK293T cells were purchased from the Cell Bank of the China Academy of Sciences (Shanghai, China). Four target sequences (Table S1) were inserted into lenticrispr v2 and transfected HEK293T cells to produce a lentivirus for the generation of ATF3 knockout cells. The HT1080 cells were infected with the lentivirus for 48 h and cultured for 48 h in DMEM containing 2 µg/mL puromycin. *ATF3*-overexpressing cells were established according to the preceding protocol. *ATF3*-overexpressing plasmids were purchased from Guang Zhou JiDan, Guangdong. All cells were cultured in Dulbecco's modified Eagle's medium (DMEM); Nanjing KeyGen Biotech, Nanjing, China) supplemented with 10% (v/v) fetal bovine serum (FBS; ExCell Biotech Co. Ltd., Taicang, China) in an incubator at 37 °C and under a 5% CO_2_ atmosphere.

### Cell viability and colony formation assays

Cell viability was measured with a CCK-8 (Dojindo Laboratories, Kumamoto, Japan). HT1080 and Hepg2 cells were cultured overnight in a 96-well plate (Yongjin, Guangzhou, China) at densities of 2 × 10^3^/well and 4 × 10^3^/well. The cells were subjected to the preceding agents for 48 h and then 90 µL fresh medium and 10 µL CCK-8 solution (EdU; 10 µM) were added to them. Optical densities (OD) were measured in a microplate reader (BioTek Instruments, Winooski, VT, USA) at 450 nm. The EdU images were obtained by fluorescence microscopy, magnification 10x (Leica, Germany). Colony formation assays were used to evaluate cell viability. About 1,000 cells were placed in six-well plates and cultured in complete DMEM. The latter was changed every 2 d. After 1 wk, the colonies were washed twice with phosphate-buffered saline (PBS), incubated with 4% (v/v) paraformaldehyde (PFA; Beyotime Biotechnology, Shanghai, China) for 30 min, and stained with crystal violet solution (Beyotime Biotechnology).

### RNA sequencing analysis

HT1080 cells were seeded into a six-well plate at a density of 4 × 10^4^/well. The following day, the cells were subjected to KCl, erastin, GSK'414, KCl + erastin, or GSK'414 + erastin for 48 h and analyzed by Su Zhou Jin Weizhi.

### Western blotting (WB)

Briefly, the cells were lysed in radioimmunoprecipitation (RIPA) buffer (150 mM NaCl, 1.0% (v/v) NP-40, 0.5% (w/v) sodium deoxycholate, 0.1% (v/v) sodium dodecyl sulfate (SDS), 50 mM Tris-HCl (pH 8.0), and Roche Complete Mini EDTA-free) for 30 min. They were then subjected to sodium dodecyl sulfate-polyacrylamide gel electrophoresis (SDS-PAGE) for WB. The following primary antibodies were used: anti-ATF3 (1:1,000; #18665; Cell Signaling Technology (CST), Danvers, MA, USA), anti-PERK (1:1,000; #5683; CST), anti-ATF6 (1:1,000; #65880; CST), and anti-IRE1α (1:1,000; #3294; CST).

### Immunohistochemistry (IHC)

Tissue microarrays were obtained from the Affiliated Hospital of Jiangsu University (Zhengjiang, China), subjected twice to xylene for 10 min each time, and hydrated with 100% (v/v), 95% (v/v), and 70% (v/v) ethanol for 5 min per step. Sodium citrate solution (pH 6.0) was used for antigen retrieval and the tissue microarrays were blocked in 5% (v/v) bovine serum albumin (BSA) for 1 h. The slides were incubated with anti-ATF3 (1:1,00; Abmart, Shanghai, China) and anti-COX2 (1:100; Abcam, Cambridge, UK) antibodies at 4°C overnight. They were then stained with secondary antibody (Aobose, Shandong, China) for 1 h, incubated with horseradish peroxidase (HRP)-conjugated Streptomyces ovalbumin (Aobose, Shandong, China) for 30min, stained with 3,3'-diaminobenzidine (DAB, Servicebio, Wuhan, China) for 5 min to monitor primary antibody levels, and finally stained with hematoxylin to disclose the nuclei.

### Xenograft tumor models

Male BALB/c-nu mice age 4-6 weeks were purchased from the Animal Center of Jiangsu University (Zhenjiang, China). All animal experiments were performed under standard conditions. HT1080 cells were cultured with or without KCl for 1 week and injected subcutaneously into the mice. When the tumor volume reached 50 mm^3^, (piperazine erastin (PE) 20 mg/kg) or dimethyl sulfoxide (DMSO) was injected every 2 day for 2 weeks and the tumors were analyzed 2 day after the final injection. Tumor volumes were calculated as shown in Eq. 1 below:

V = L × W^2^ × ½ (1)

where V is tumor volume, L is tumor length, and W is tumor width.

### Lipid peroxidation detection

Malondialdehyde (MDA) and glutathione (GSH) assay kits were purchased from Nanjing Jiancheng Bioengineering Institute, Nanjing, Jiangsu, China. A reactive oxygen species (ROS) detection kit was purchased from Dalian Meilunbio, Dalian, China. Cells were cultured in 100-mm plates (Yongjin, Guangzhou, China) and subjected to 35 mM K^+^, 3µM GSK'414, and 2.5 µM erastin for 48 h and subjected to MDA, GSH, and ROS detection.

### Transmission electron microscopy analysis

HT1080 cells were seeded into a six-well plate at a density of 4 × 104/well. The following day, the cells were subjected to KCl, erastin, GSK'414, KCl + erastin, or GSK'414 + erastin for 48 h. Then, fixed with glutaraldehyde, and analyzed by Nangjing Shiyanjia Lab.

### Statistical analysis

All data were normalized as means ± standard error of the mean (SEM). Significant differences between groups were identified by one-way analysis of variance (ANOVA) and Student's *t*-test. A value of *p* < 0.05 reflected a statistically significant difference.

## Results

### A high-potassium environment confers ferroptosis resistance in cancer cells

**Figure 1:**
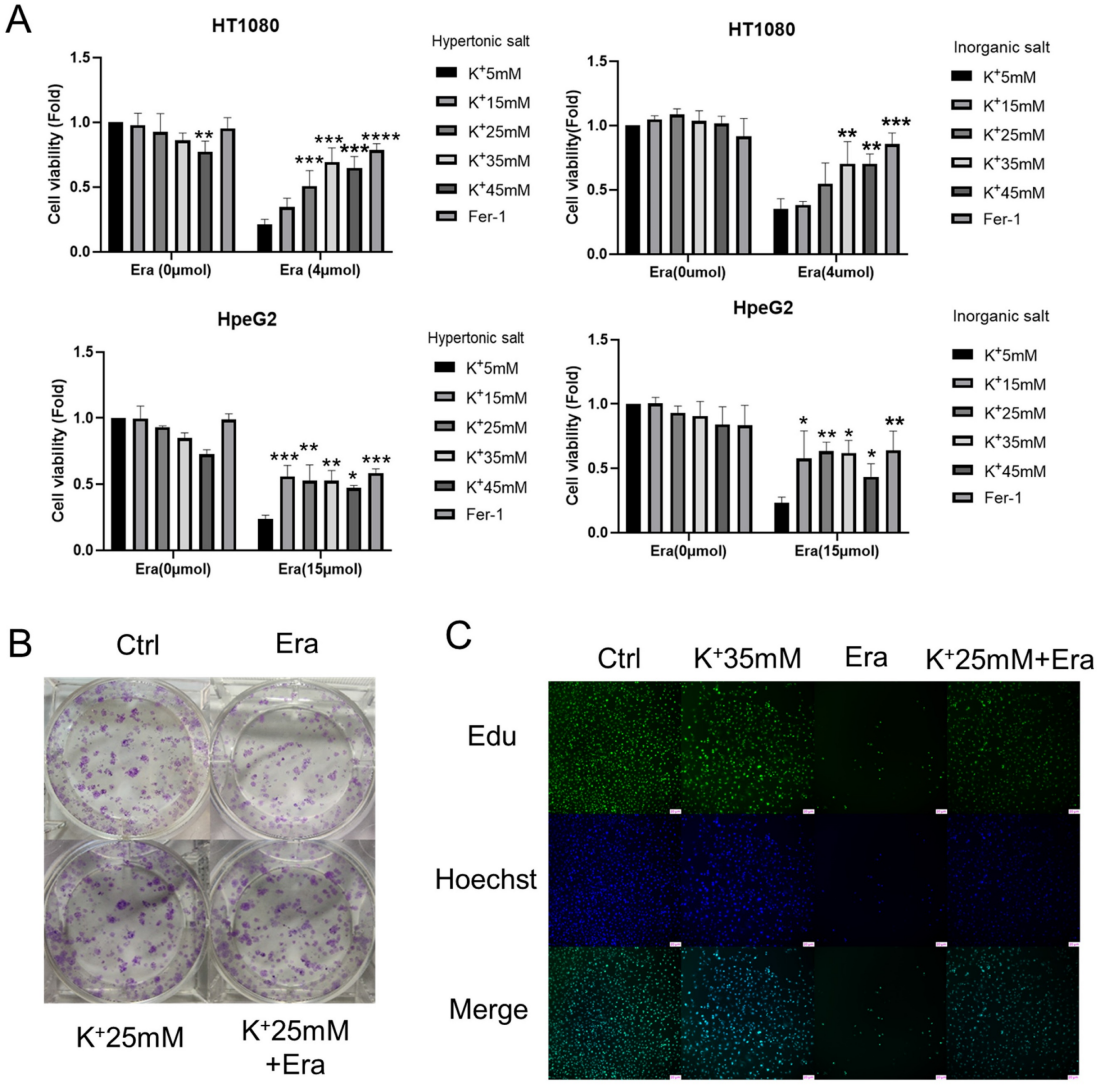

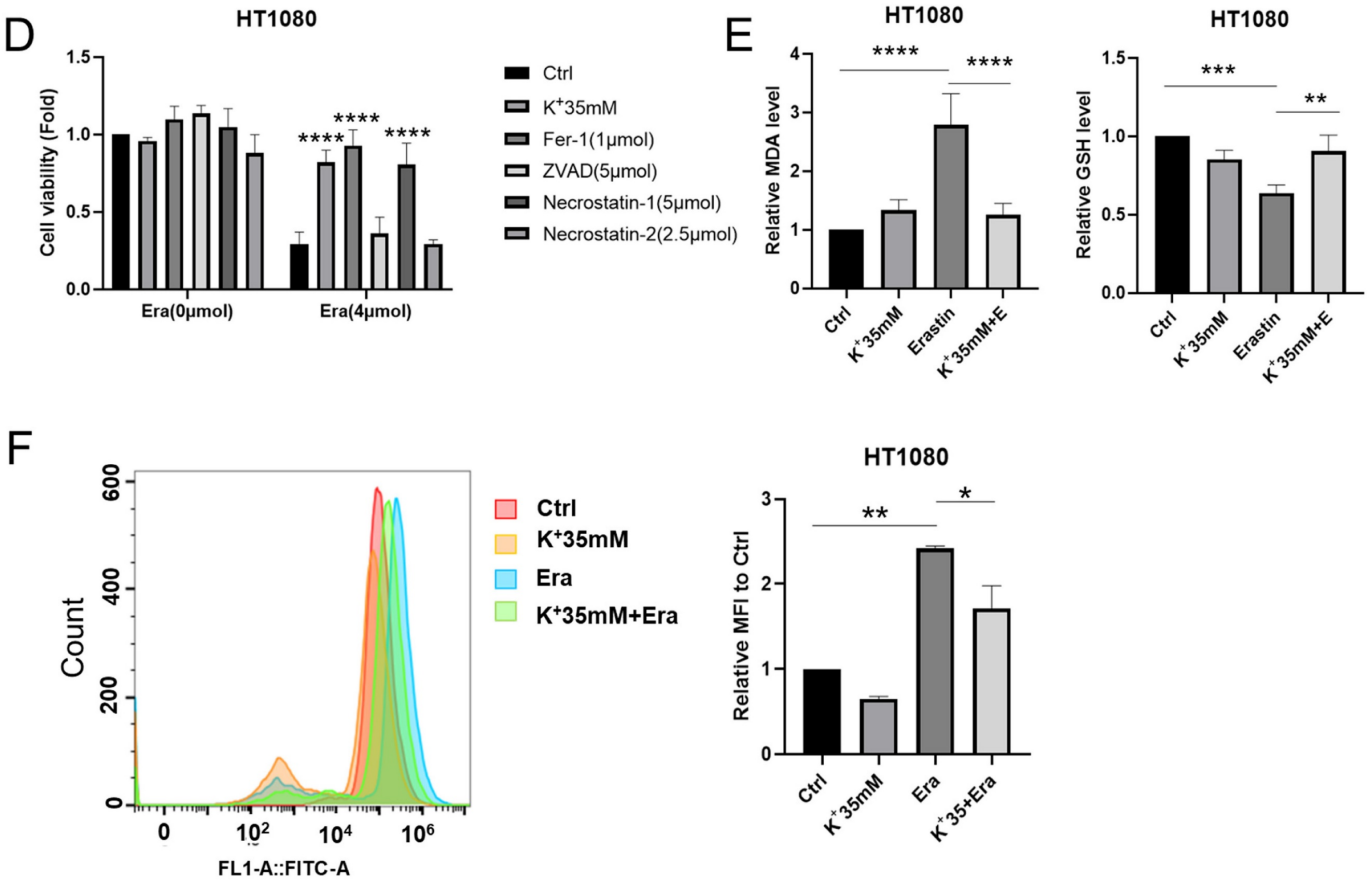
** High potassium environment confers cancer cells resistance to ferroptosis. (A)** CCK-8 assay for cell viability of HT1080 and HepG2 cells treated with Erastin (4 µmol, 15 µmol) in a high potassium environment for 48 h, respectively. The relative viability was normalized to the K^+^ 5mM group. **(B)** Colony-forming ability of HT1080 cells cultured with K^+^ 25mM medium and Erastin (1 µmol) for one week. **(C)** HT1080 cells were cultured with K^+^ 35mM medium and Erastin (4 µmol) in 96 wells for 48 h, then incubated with 10 µmol Edu. Images were obtained through a fluorescence microscope.** (D)** The cell viability of HT1080 with the treatments DMSO, Erastin (4 µmol) for 48 h combined with Ferrostain-1 (1 µmol), Z-VAD-FMK (5 µmol), Necrostatin-1(5 µmol) and Necrostastin-2 (2.5 µmol) were monitored using a CCK-8 assay. The relative viability was normalized to K^+^ 5 mM group. **(E-F)** MDA, GSH, and ROS levels in HT1080 cell treated with Erastin (2.5 µmol) in a high potassium environment for 48 h. ∗P< 0.05. ∗∗P< 0.01. ∗∗∗P< 0.001. ∗∗∗∗P< 0.0001.

We set up hyperosmotic and isotonic potassium concentrations to assess the relationships between high-potassium conditions and ferroptosis. The CCK-8 assay revealed that 35 mM potassium cation significantly reversed the ferroptosis induced by erastin but not that which was induced by RSL3 in HT1080 and HepG2 cells (Figures 1A and S1A). In contrast, sodium supplementation failed to rescue ferroptosis (Figure S1B). The colony formation assay and EdU incorporation revealed that potassium-treated cells proliferated in the presence of erastin compared to the erastin control (Figures 1B, 1C, and S1C). Necrostastin-1 suppressed necroptosis by inhibiting receptor-interacting serine/threonine-protein kinase 1 (RIPK1). However, Hanna Yuk showed that necrostastin-1 also reversed erastin-induced ferroptosis. In the present work, we used necrostastin-2 for the express purpose of inhibiting necroptosis 12. The CCK-8 assay confirmed that whereas neither carbobenzoxy-valyl-alanyl-aspartyl-[O-methyl]-fluoromethylketone (Z-VAD-FMK) nor necrostastin-2 regulated ferroptosis, extra K^+^, necrostatin-1, and Fer-1 significantly prevented ferroptosis after erastin treatment (Figures 1D and S1D). MDA, GSH, and ROS flow cytometry were also used to reveal ferroptosis. In response to erastin treatment, the potassium-treated cells had lower MDA and ROS and higher GHS levels than the normal cells (Figures 1E, 1F, and S1E). The preceding results suggest that a high-potassium environment plays a critical role in ferroptosis induction.

### A high-potassium environment is associated with ER stress

**Figure 2:**
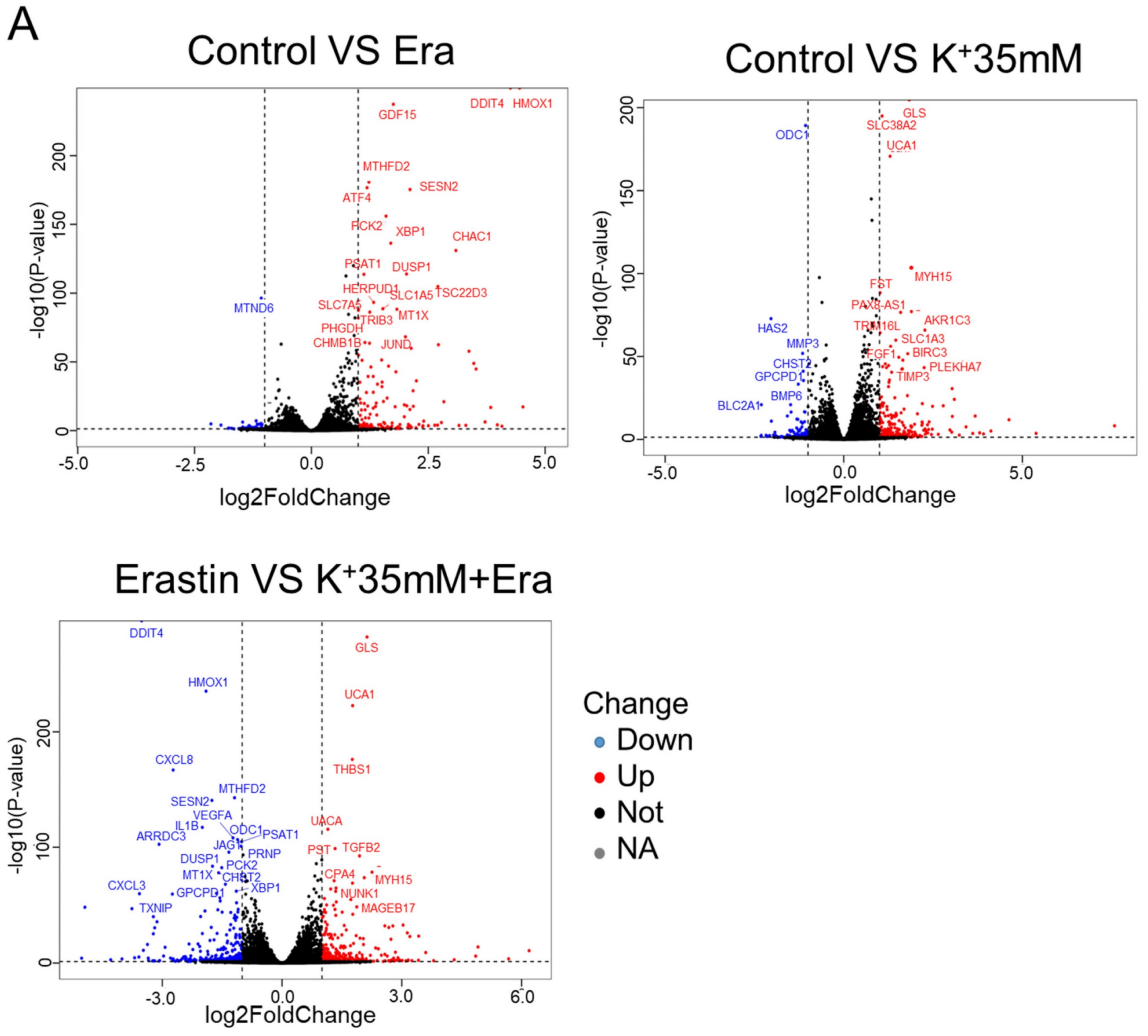

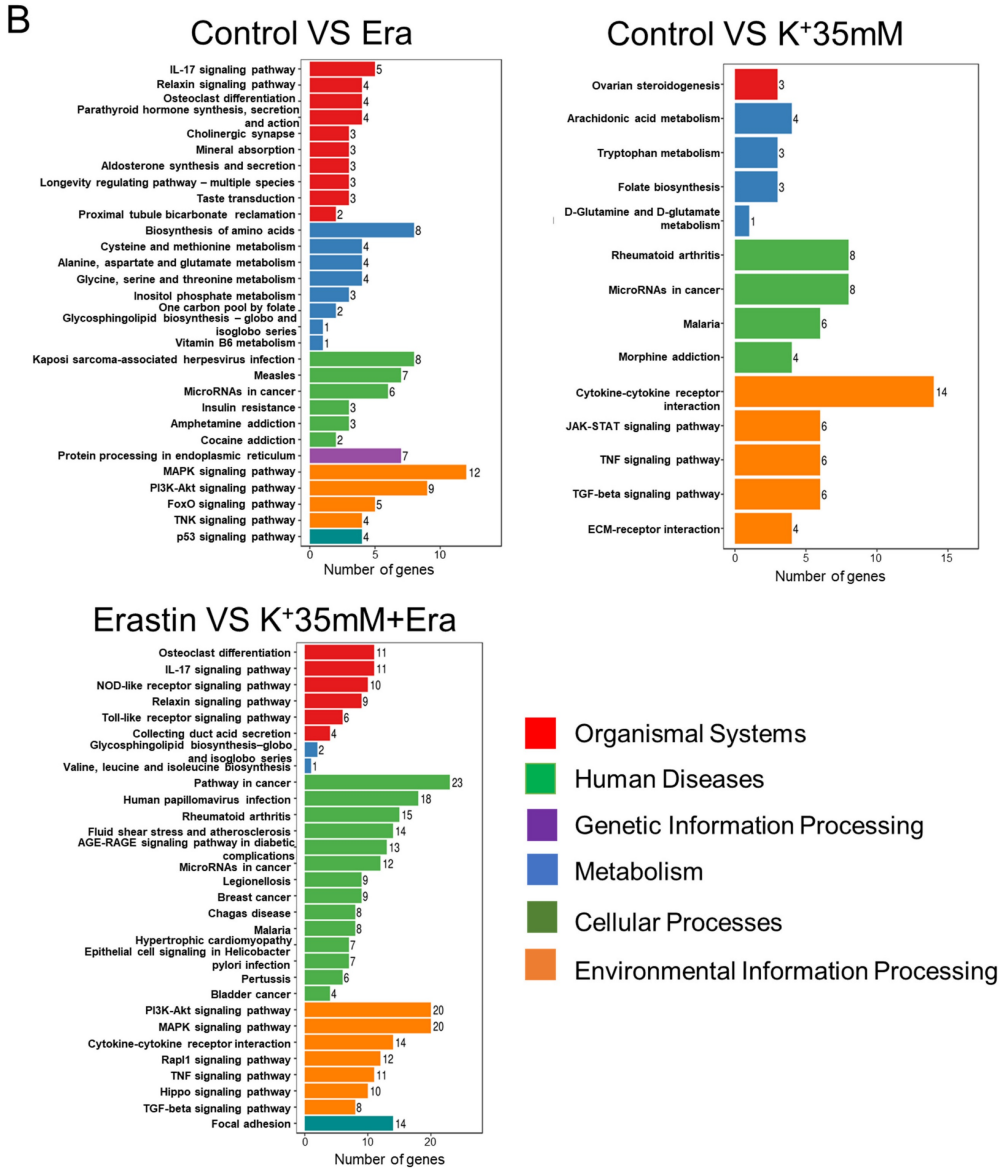

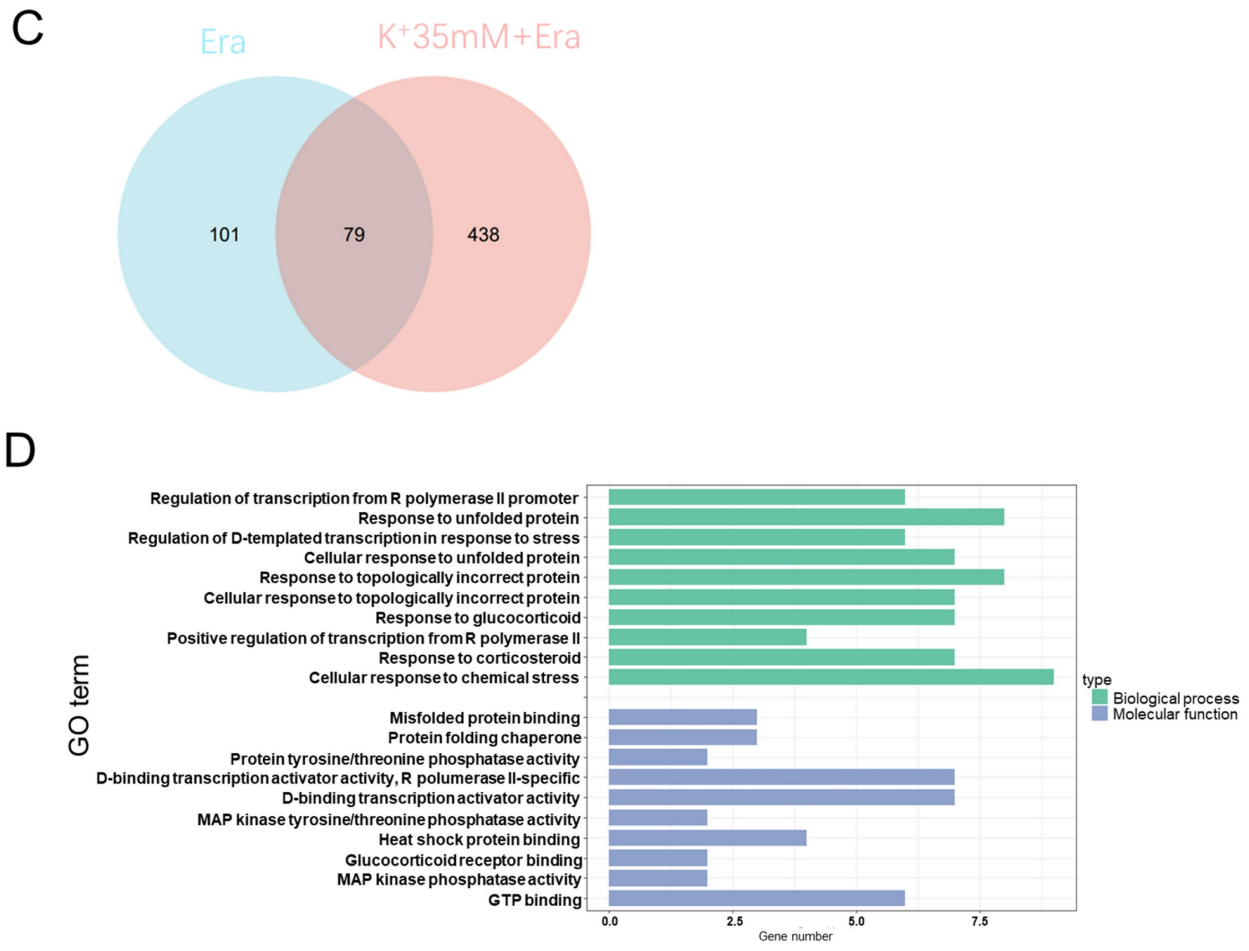
** RNA sequencing indicated that a high potassium environment is associated with ER stress. (A-B)** Volcano plot and KEGG analysis of RNA sequencing data from HT1080 cells treated with K^+^ 35mM, Erastin, and a combination of K^+^ 35mM and Erastin. **(C-D)** Venn diagram and GO analysis of Erastin group and K^+^ 35mM+Erastin group.

RNA sequencing detected changes in cellular RNA level following treatment with 5 mM K^+^, erastin, 35 mM K^+^, or 35 mM K^+^ + erastin to identify correlations between a high-potassium environment and ferroptosis. The results revealed that high extracellular potassium significantly modulated amino acid metabolism. The RNA levels of glutaminase (GLS) and the amino acid transporters SLC38A2, SLC2A12, and SLC1A3 were significantly increased. The KEGG and GO analyses disclosed that the high-potassium treatment significantly altered the cytokine-cytokine receptor interaction pathway (Figures 2A, 2B, and S2B). The results of both the present study and previous investigations showed that the erastin treatment upregulated ER-related genes 13. The expression levels of *DDIT4, HMOX1, ATF4, XBP1, HERPUD1*, and* ATF3* were substantially higher for the erastin treatment than for the control. The KEGG and GO analyses confirmed significant alteration of the ER pathway. Nevertheless, additional extracellular potassium significantly decreased the RNA levels of *HMOX1, DDIT4, TXNIP*, and* XBP1* (Figures 2A, 2B, S2A, and S2C). To elucidate the mechanisms underlying these responses, we analyzed the foregoing genes and found that the expression levels of 79 of them had changed in the elastin and combination groups relative to the control (Figure 2C). The GO analysis associated these genes with the terms “molecular function”, “cellular component”, and “biological process”. Analyses of the terms “biological process” and “molecular function” revealed that the genes were involved mainly in misfolded protein binding associated with ER stress (Figure 2D).

### A high-potassium environment alleviates erastin-induced ER stress

**Figure 3:**
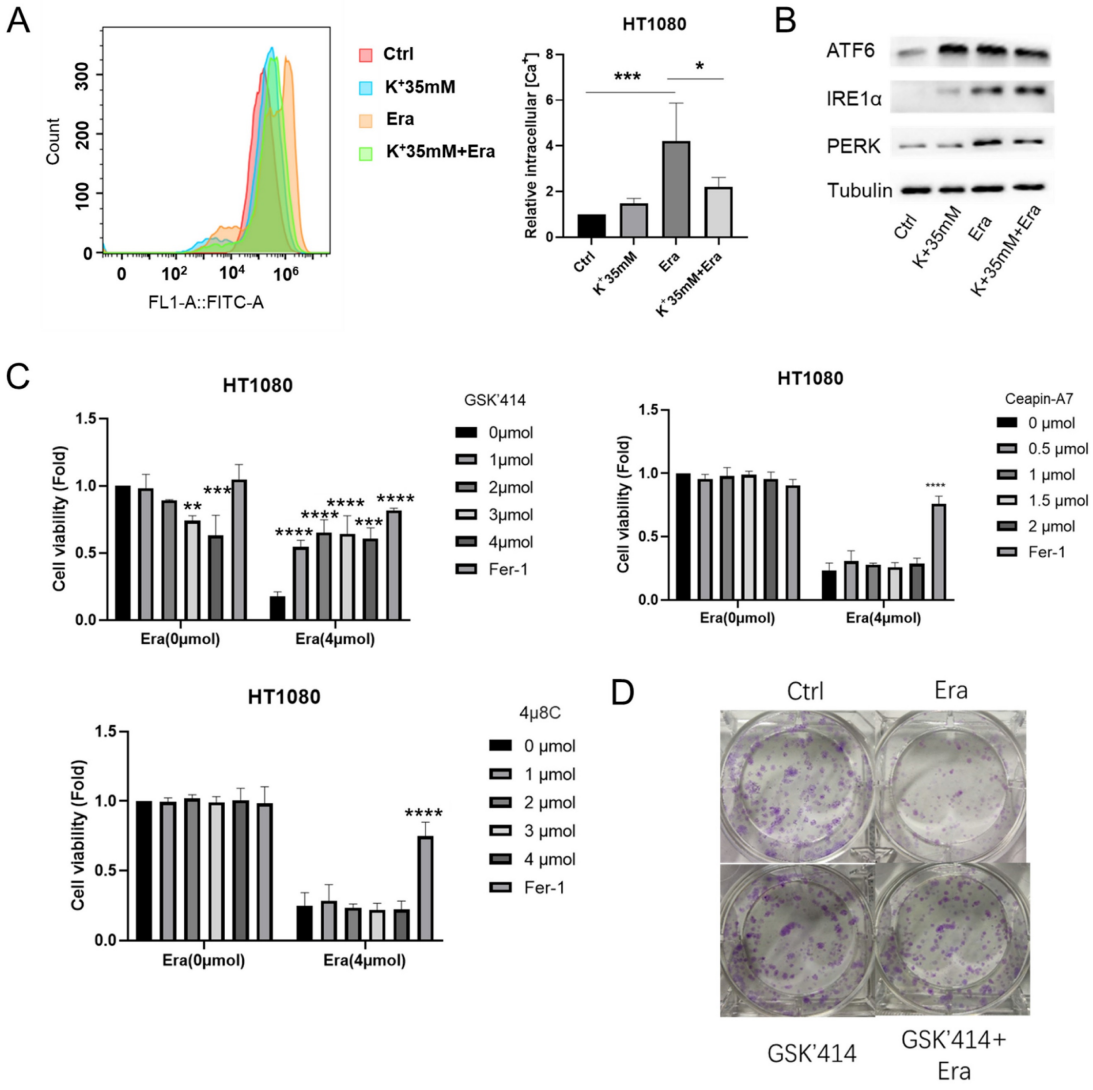

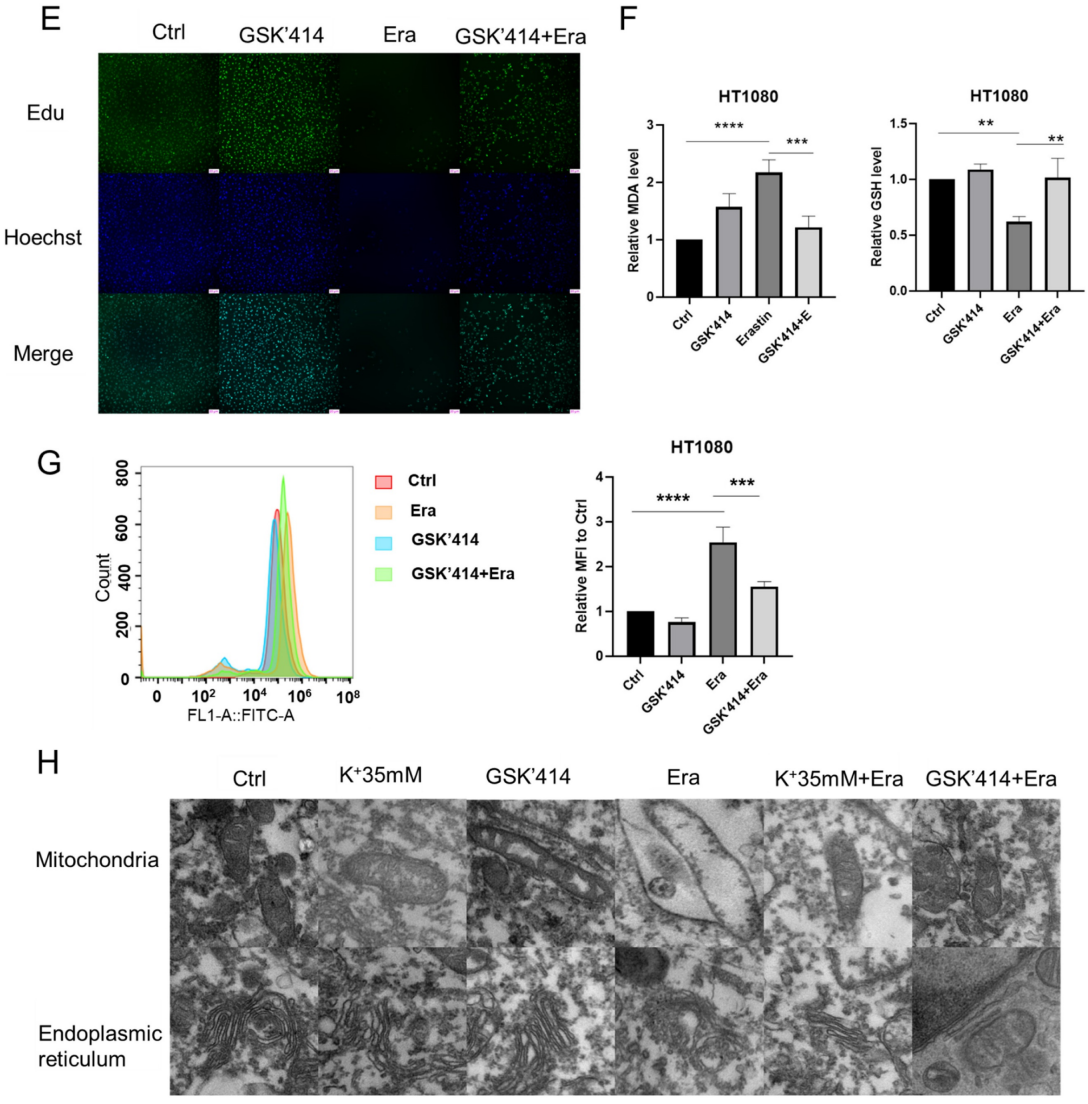
** High potassium environment relieved ER stress induced by Erastin. (A)** Intracellular Ca2^+^ level of HT1080 treated with Erastin (2.5 µmol) in a high potassium environment for 48 h. **(B)** The expression level of ER-associated genes ATF6, IRE1α, and PERK. **(C)** CCK-8 assay assessed the cell viability of HT1080 treated with Erastin (4 µmol) combination of GSK'414, Ceapin-A7, and 4u8C for 48 h, respectively. The relative viability was normalized to control. **(D)** Colony forming ability of HT1080 cells cultured with GSK'414 (2 µmol) and Erastin (1 µmol) for one week. **(E)** HT1080 cells were cultured with GSK'414 (2 µmol) and Erastin (4 µmol) for 48 h, then incubated with 10 µmol Edu. **(F-G)** MDA, GSH, and ROS levels in HT1080 cells treated with Erastin (2.5 µmol) and GSK'414 (2 µmol) for 48 h. **(H)** TEM analysis of mitochondrion and endoplasmic reticulum of HT1080 cells treated with K^+^ 35 mM, GSK'414, and Erastin.

DaeYong Lee confirmed that perturbation of intracellular potassium homeostasis induced severe ER stress and apoptosis 14. However, the optimal balance between extracellular potassium and ER stress has not yet been identified. Hence, we applied Fura-3, AM and measured the intracellular Ca^2+^ level as an ER stress marker. A high-potassium environment drastically reduced intracellular Ca^2+^ accumulation following erastin treatment (Figure 3A). In humans, three ER transmembrane proteins (PERK, IRE1α, and ATF6) operate as ER stress sensors 15. Here, a high-potassium environment and erastin treatment upregulated IRE1α and ATF6. However, the expression levels of PERK and ATF6 were not higher in the combination treatment than in the erastin treatment (Figure 3B).

We then inhibited PERK, ATF6, and IRE1α with GSK2606414 (GSK'414), ceapin-A7, and 4u8C, respectively 16-18. The CCK-8 and colony formation assays and EdU incorporation revealed that only the PERK inhibitor GSK2606414 significantly reversed erastin-induced ferroptosis (Figures 3C-3E). The MDA, GSH, and ROS levels confirmed this reversal. The GSK'414 treatment decreased the MDA and ROS levels and increased the GSH level following the erastin treatment (Figures 3F and 3G). Transmission electron microscopy disclosed that a high-potassium environment and GSK'414 reversed erastin-induced mitochondrial and ER damage (Figure 3H). The preceding results imply that a high-potassium environment reverses ferroptosis through the PERK pathway.

### High potassium inhibited ferroptosis rescue by regulating *ATF3* expression

**Figure 4:**
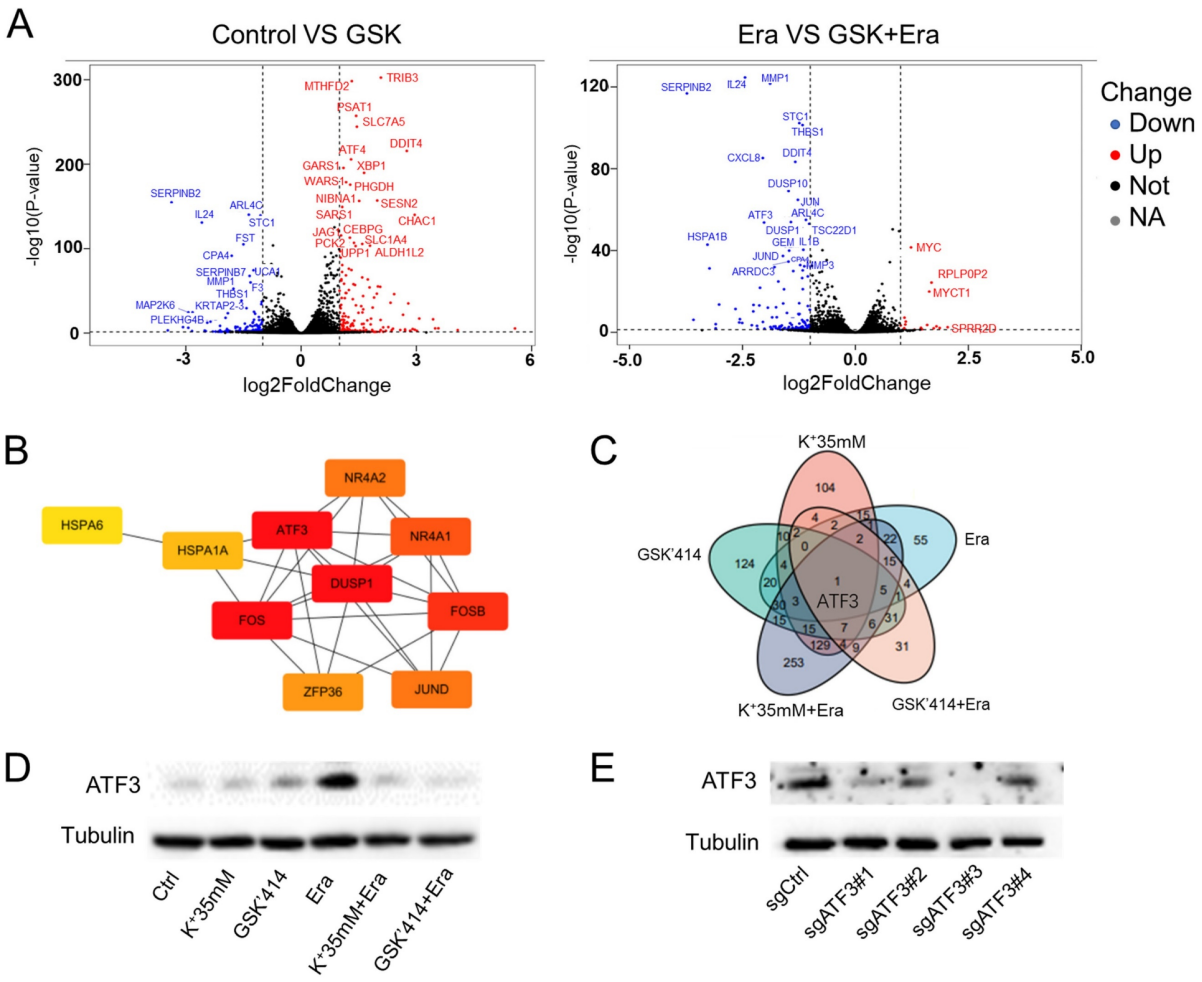

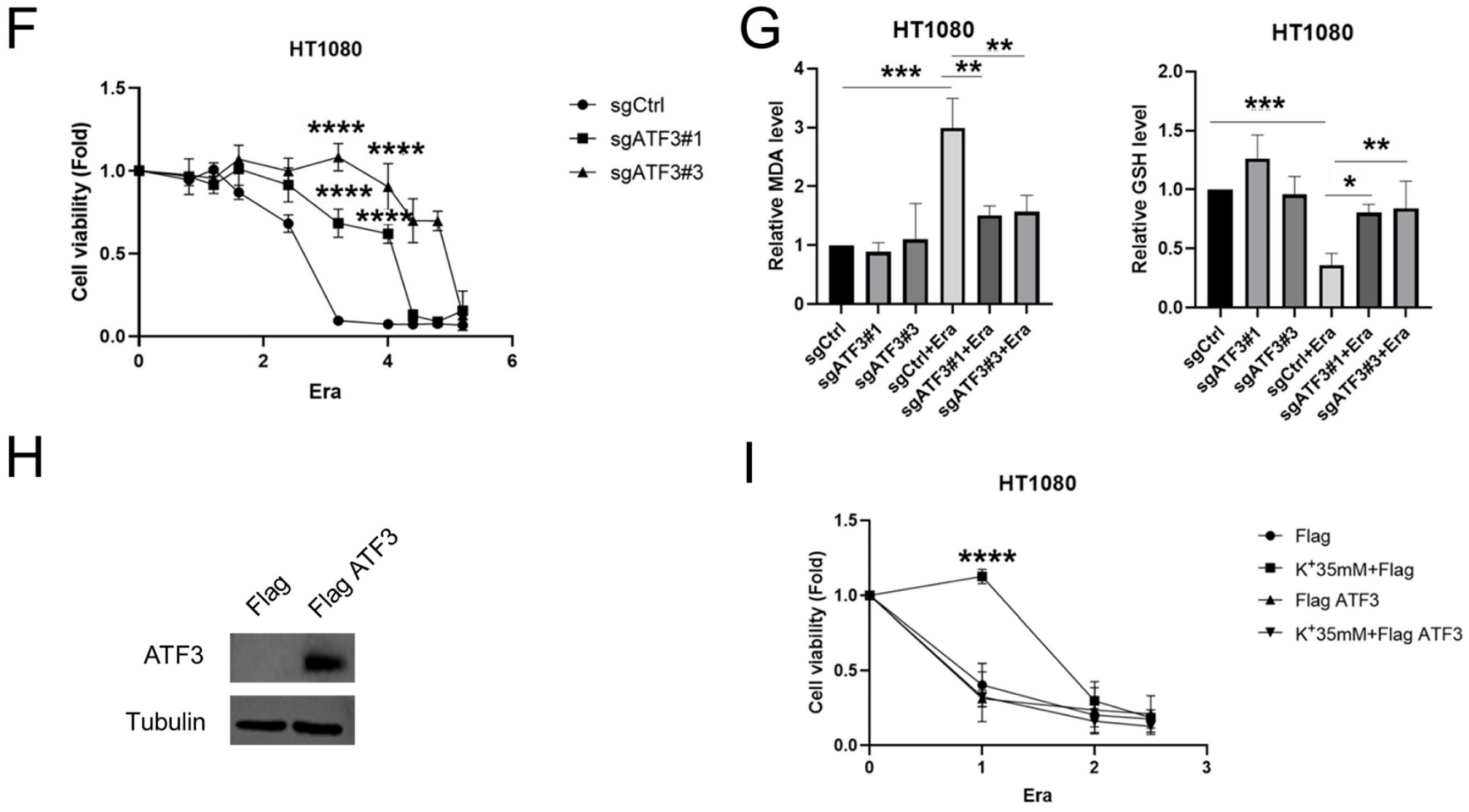
** ATF3 plays a significant role in ferroptosis in a high potassium environment. (A)** Volcano plot analysis of RNA sequencing results of HT1080 treated with GSK'414 and a combination of GSK'414 and Erastin. **(B)** Hub genes analysis of Erastin, K^+^ 35mM+Erastin, GSK'414+Erastin. **(C)** Venn diagram analysis indicated that ATF3 played a vital role in ferroptosis. **(D)** The expression level of ATF3 when treated with K^+^35mM, GSK'414 (3µmol), and Erastin (2.5 µmol) for 48 h. **(E-G)** Knockdown ATF3 attenuated sensitivity to ferroptosis and MDA, confirmed by the GSH level. **(H-I)** Overexpression ATF3 enhanced sensitivity to ferroptosis.

We then performed RNA sequencing on the GSK'414-treated cells to identify the factors explaining the rescue of ferroptosis mediated by a high-potassium environment. The ER-related proteins DDIT4, ATF3, XBP1, and HSPA1B were upregulated in response to GSK'414 but downregulated following the erastin combination treatment (Figure 4A). The KEGG analysis showed that the GSK'414 treatment and the erastin combination affected mainly the phosphatidylinositol-3-kinase (PI3K)-protein kinase B (Akt) and mitogen-activated protein kinase (MAPK) signaling pathways, respectively (Figures S3A and S3B). The GO analysis demonstrated that the GSK'414 treatment influenced the PERK-mediated unfolded protein response while the erastin combination was related mainly to the inflammatory response (Figures S3C and S3D).

We analyzed the foregoing genes to identify the factors playing crucial roles in reversing cell ferroptosis. The relative expression levels of these genes changed in the erastin, 35 mM K^+^ + erastin, and GSK'414 + erastin groups. Twenty-four genes were altered in all three groups and *ATF3*, *DUSP1*, *FOS*, and *FOSB* might be the hub genes regulating ferroptosis (Figures 4B, S3E, and S3F). Nevertheless, RNA sequencing of the 35 mM K^+^- and GSK'414-treated cells indicated that *ATF3* might be the pivotal gene (Figure 4C).

HT1080 cells were treated with high potassium concentrations, GSK'414, and erastin for 2 days in an attempt to clarify the relationship between *ATF3* in ferroptosis and a high-potassium environment. WB demonstrated that erastin significantly upregulated *ATF3* whereas the combination of 35 mM K^+^ or GSK'414 plus erastin downregulated *ATF3* to the expression level of the control (Figure 4D). We used the CRISPR/Cas9 tool to generate *ATF3* knockout HT1080 cells and corroborate the preceding results. We constructed four sgRNA sequences and observed that both sgATF3#1 and sgATF3#3 successfully silenced ATF3 (Figures 4E and S3G). The CCK-8 assay revealed that *ATF3* knockout prevented ferroptosis after the 4 µM erastin treatment (Figure 4F). The MDA and GSH measurements confirmed that *ATF3* knockout reversed ferroptosis-induced damage. *ATF3* knockout substantially decreased the MDA and increased the GSH levels relative to those of the erastin-treated control (Figure 4G). We also overexpressed *ATF3* to verify that a high-potassium environment reverses ferroptosis by inhibiting *ATF3* (Figure 4H). High potassium-mediated ferroptosis repression under the condition of *ATF3* overexpression indicates that other heretofore unidentified factors are also involved in ferroptosis. However, the CCK-8 assay showed that a high-potassium environment could not reverse ferroptosis in response to *ATF3* overexpression in the presence of erastin (Figure 4I). These results confirmed that a high-potassium environment inhibits ferroptosis by repressing *ATF3*.

### Cells subjected to high potassium prevented ferroptosis *in vivo*

**Figure 5:**
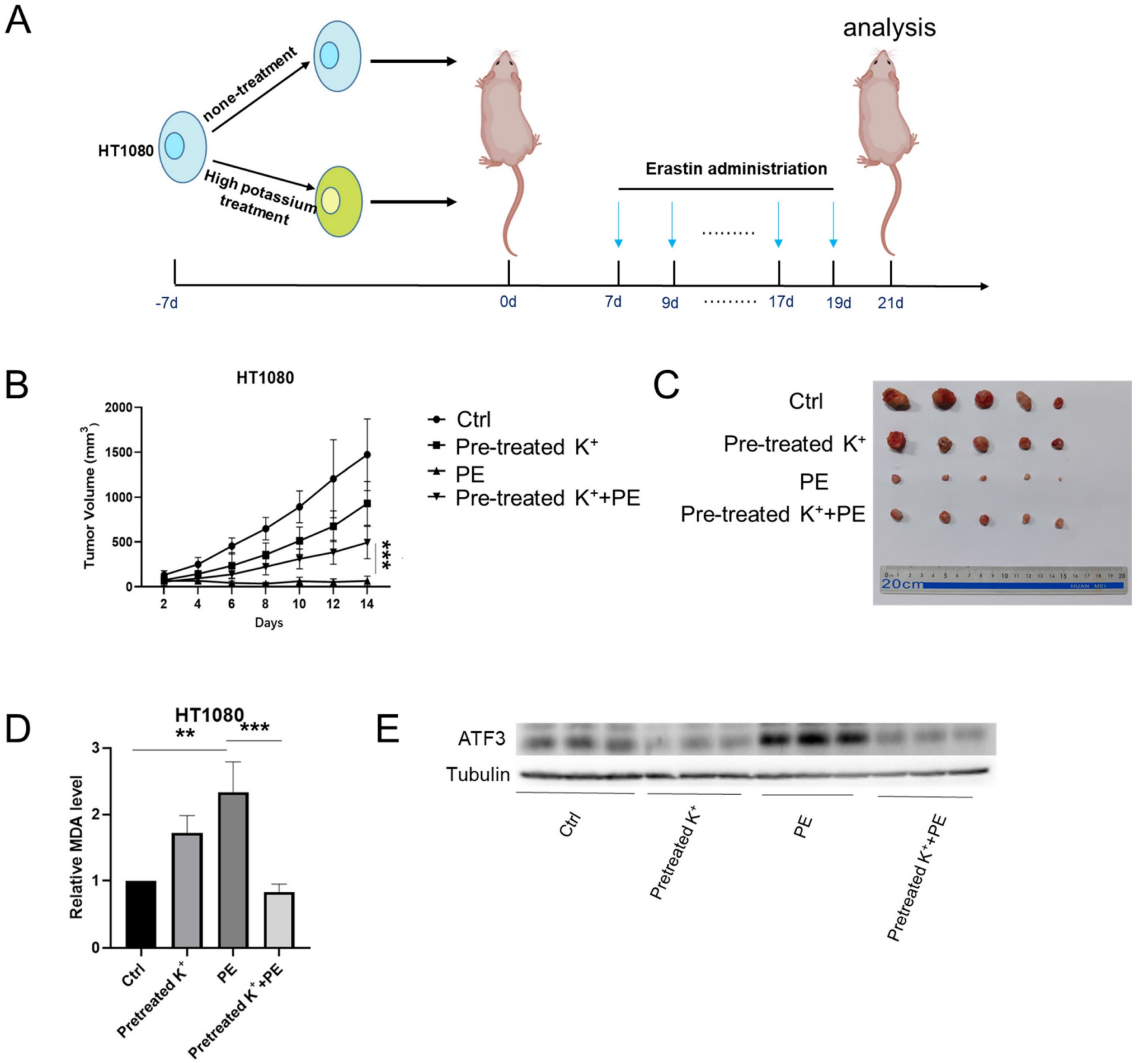

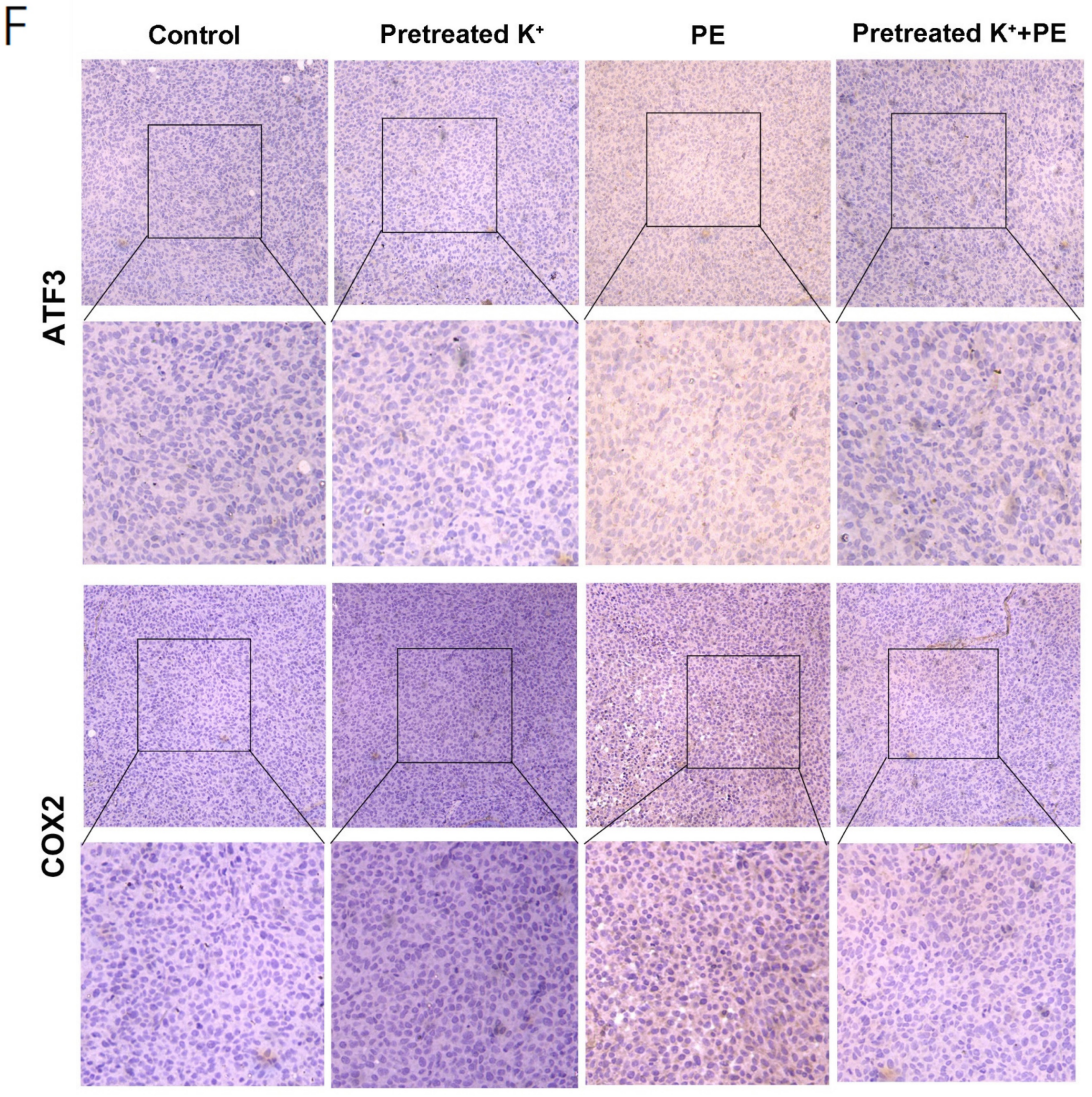
** High potassium-treated cells prevented ferroptosis *in vivo*. (A)** Schematic illustration of the therapeutic scheme *in vivo*. HT1080 cells were pretreated with or without KCL (35 mM) for one week and then injected subcutaneously into BALB/c-nu mice. The mice were treated with Piperazine Erastin (10 mg/kg/s.c.) by peritumoral injection every other day for two weeks. **(B-C)** The tumor image of each group taken at the end of the study and tumor volumes in mice indicated treatment. **(D)** MDA level in the indicated tumor tissues.** (E-F)** Western blotting and IHC images of COX2 and ATF3 in indicated treatment mice tumors.

We subcutaneously injected HT1080 cells into BALB/c-nu mice to validate the observation that a high-potassium environment prevents ferroptosis* in vitro*. The mice were peritumorally injected with KCl and PE when the tumor volume reached 50 mm^3^. However, the mice died following the KCl injection. Eil reported abundant cancer cells in a high-potassium environment (Figure 5A) 4. Here, we cultured HT1080 cells with or without KCl for 1 week and then subcutaneously injected them into BALB/c-nu mice. We injected PE every 2 d for 2 weeks and measured the tumors 2 days after the final injection.

Compared with the control, the KCl pretreatment and PE treatment mildly and strongly inhibited tumor growth, respectively. Nevertheless, PE treatment following KCl pretreatment protected the cells against ferroptosis (Figures 5B and 5C). The MDA measurements confirmed that KCl pretreatment alleviated PE-induced ferroptosis (Figure 5D). WB disclosed that KCl pretreatment followed by PE treatment downregulated *ATF3* to a greater extent than the PE treatment alone (Figure 5E). The IHC showed that the levels of *ATF3* and the ferroptosis indicator COX2 were significantly lower in the combination group than in the PE group (Figure 5F). Taken together, the preceding results indicated that a high-potassium environment protects cells against ferroptosis.

## Discussion

It was previously shown that ionic homeostasis in general, and potassium homeostasis in particular, regulate apoptosis. Increasing the extracellular K^+^ concentration or inhibiting K^+^ efflux via channel blockers suppresses cellular apoptosis 5, 19, 20. However, it is unknown whether K^+^ also influences other types of cell death such as ferroptosis. The latter is iron-dependent regulated cell death and it morphologically and biochemically differs from apoptosis 21, 22. Several studies have implied that various ions including Fe^2+^, Ca^2+,^ and Zn^2+^ are implicated in ferroptosis 21, 23-25. The present study demonstrated that a high-potassium environment seems to prevent ferroptosis in cancer cells.

Mitochondria, ER, lysosomes, lipid droplets, peroxisomes, nuclei, the Golgi apparatus, and other subcellular organelles are involved in ferroptosis. They generate signals driving lipid biosynthesis, iron accumulation, and lipid peroxidation 26. The present work revealed that a high-potassium environment alleviates ferroptosis through ER stress. Several investigations have shown that the TME is significantly involved in ER stress, hypoxia, low pH/acidosis, and nutrient deprivation 27-29. Adaptive ER stress favors cellular survival and reprogramming. Persistent, robust ER stress may induce cell death. As ER stress is higher in cancer cells than normal ones, it is promising as a target for anticancer agents. However, the present study revealed that a high-potassium environment might alleviate induced ER stress. Hence, future studies should endeavor to explore whether high extracellular K^+^ affects the therapeutic efficacy of drugs targeting the ER. Previous research also disclosed that high extracellular K^+^ levels may influence mitochondrial metabolism 30. Nevertheless, our RNA sequencing results failed to corroborate this finding. Therefore, it remains to be determined whether high K^+^ concentrations affect other subcellular organelles.

Ferroptosis also regulates neurodegenerative conditions such as Alzheimer's disease (AD) and Parkinson's disease (PD) 31, 32. Though these disorders have imposed substantial socioeconomic and healthcare burdens, there are few therapeutic strategies for them. Several studies have reported that elevated oxidative and ER stress comprise the main molecular mechanisms of neurodegenerative diseases. Prior research reported that secondary increases in calcium ion levels also lead to ferroptosis 24, 33, 34. Other earlier work demonstrated that PERK inhibitors prevented neurodegeneration in an animal PD model. However, these drugs could also cause secondary pancreatic toxicity 35. The present study confirmed that the PERK inhibitor GSK'414 protected cells against ferroptosis and demonstrated that high K^+^ concentrations attenuate ER stress and inhibit increases in Ca^2+^. Future investigations should aim to determine whether elevated cerebral K^+^ concentrations critically regulate neurodegenerative diseases.

## Supplementary Material

Supplementary figures and table.

## Abbreviations

KEGG: Kyoto Encyclopedia of Genes and Genomes
GO: Gene Ontology
ER: Endoplasmic Reticulum
GSK'414: GSK'2606414
PERK: PRKR-like ER kinase
IRE1α: inositol-requiring enzyme 1α
ATF6: activating transcription factor 6
ATF3: activating transcription factor 3
