# Supplementary material for: PERK/ATF3-Reduced ER Stress on high potassium environment in the suppression of tumor ferroptosis
Source: J Cancer. 2023 May 15;14(8):1336–49. doi: 10.7150/jca.83556 (PMC10240662; doi:10.7150/jca.83556)

Table S1 Primers for knockdown plasmids

Gene	Sequence
**sgATF3#1**	**5'-CACCGGGTGTCCATCACAAAAGCCG-3'**
	**3'-AAACCGGCTTTTGTGATGGACACCC-5'**
**sgATF3#2**	**5'-CACCGCTGAGCCCGGACAATACACG-3'**
	**3'-AAACCGTGTATTGTCCGGGCTCAGC-5'**
**sgATF3#3**	**5'-CACCGCTGAGCCCGGACAATACACG-3'**
	**3'-AAACCGTGTATTGTCCGGGCTCAGC-5'**
**sgATF3#4**	**5'-CACCGCCACCGGATGCCTCTGCGC-3'**
	**3'-AAACGCGCAGAGGCATCCGGTGGC-5'**

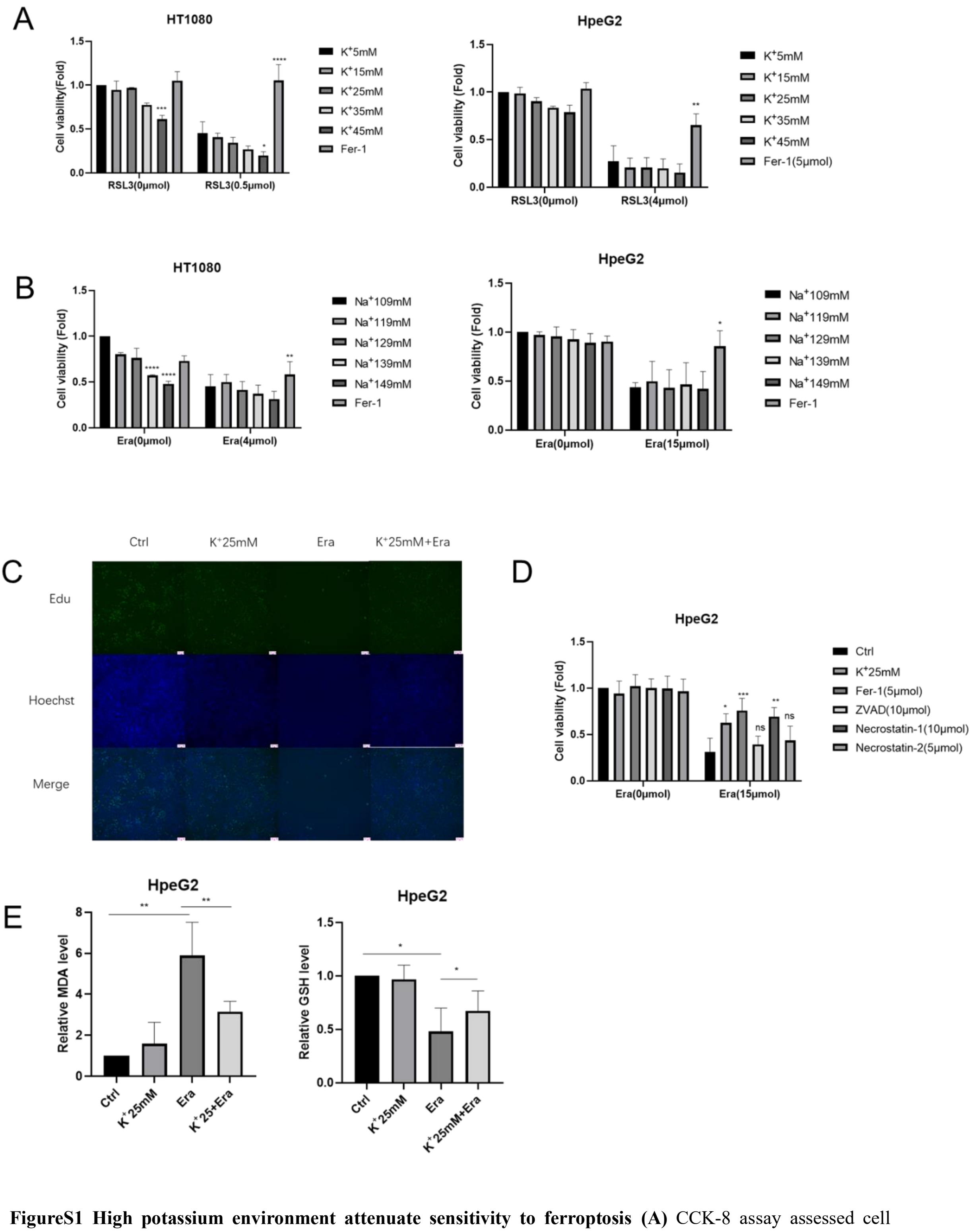

FigureS1 High potassium environment attenuate sensitivity to ferroptosis (A) CCK-8 assay assessed cell viability of HT1080 and HepG2 cells treated with RSL3 (0.5μM, 4μM) in high potassium environment for 48 h respectively. The relative viability was normalized to K^+5mM group **(B)** The viability of cells treated with Erastin in the condition of additional NaCl. **(C)** HepG2 cells cultured with K^+25mM medium and Erastin (15μM) in 96 wells-plate for 48 h then incubated with 10μM Edu. **(D)** The cell viability of HT1080 with the treatments DMSO, Erastin (15μM) for 48 h combined with Ferrostain-1 (5μM), Z-VAD-FMK (10μM), Necrostatin-1(10μM) and Necrostastin-2 (5μM) were monitored using a CCK-8 assay. (E) MDA and GSH level in HT1080 cells treated with Erastin (10μM) in high potassium environment for 48 h. *P < 0.05. **P < 0.01. ***P < 0.001. ****P < 0.0001.

A

B

C

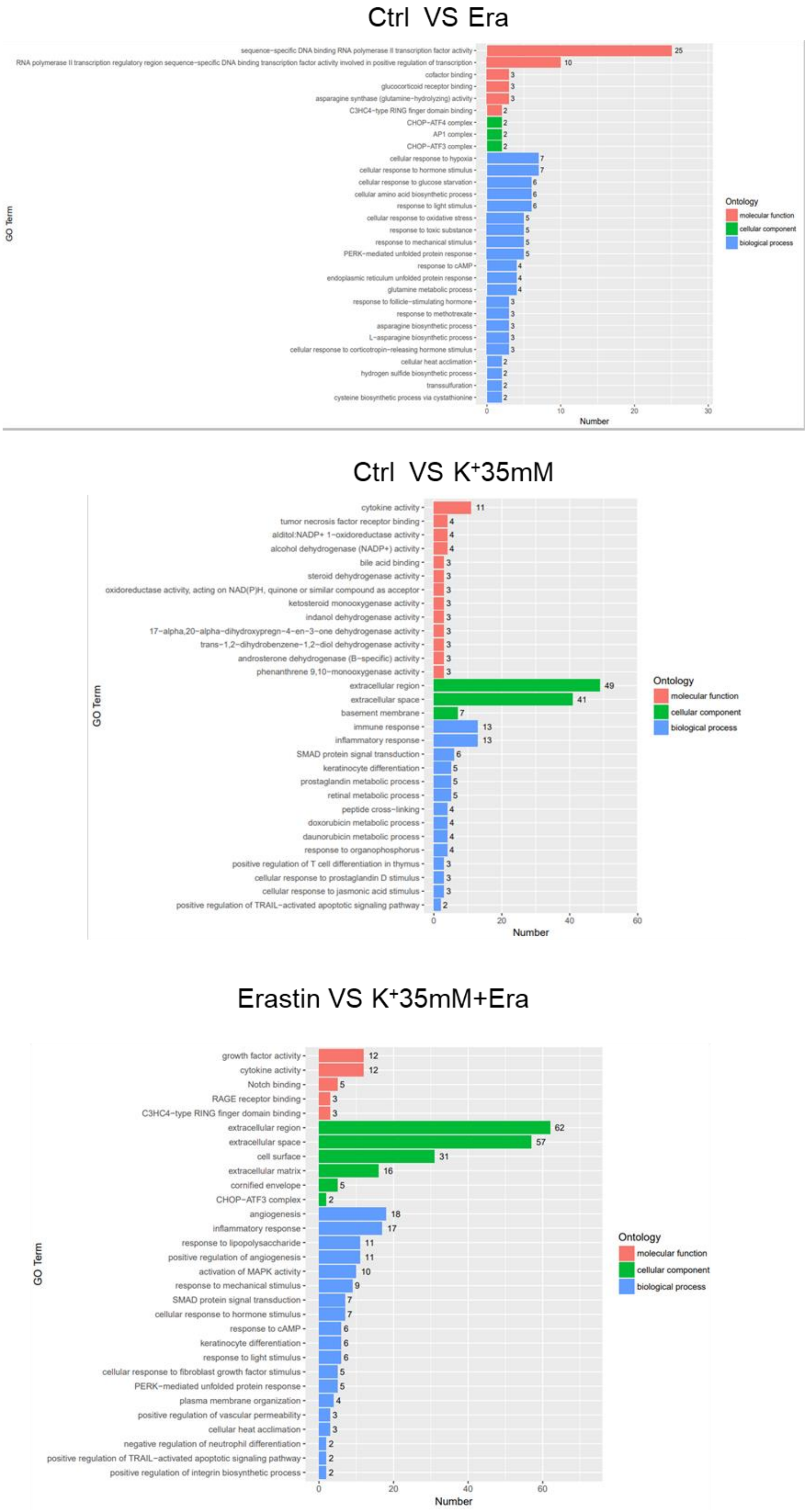

FigureS2 GO analysis indicated high potassium environment may regulate PERK pathway (A-C) The analysis of GO which cells treated with Erastin, K^+35mM and combination of K^+35mM and Erastin.

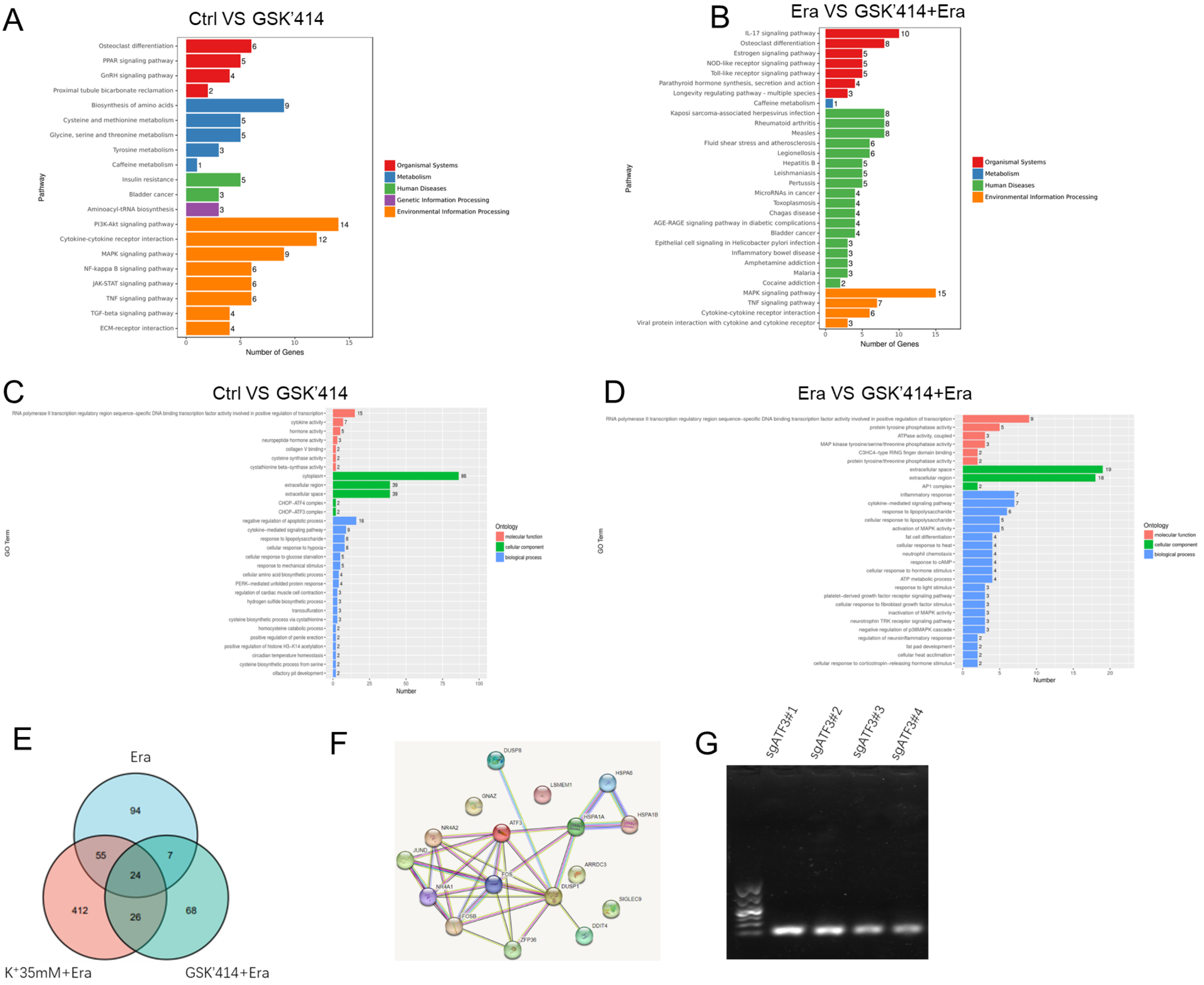

FigureS3 The RNA sequence analysis of cells treated with GSK'414 (A-D) The KEGG and GO analysis of cells treated with GSK'414 and combination of GSK'414 and Erastin. **(E-F)** Venn and PPI analysis of Era group, K^+35mM+Era group and GSK'414+Era group. **(G)** The verification of knockout plasmids of ATF3

Both uncropped exposure and white pictures were offered as follow:

Figure 3B

β-Tubulin

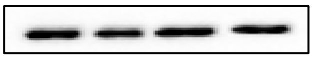

55KD

PERK

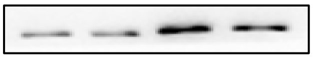

140KD

ATF6

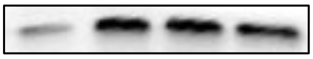

55-100KD

IRE1α

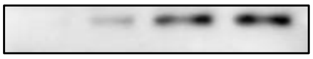

130KD

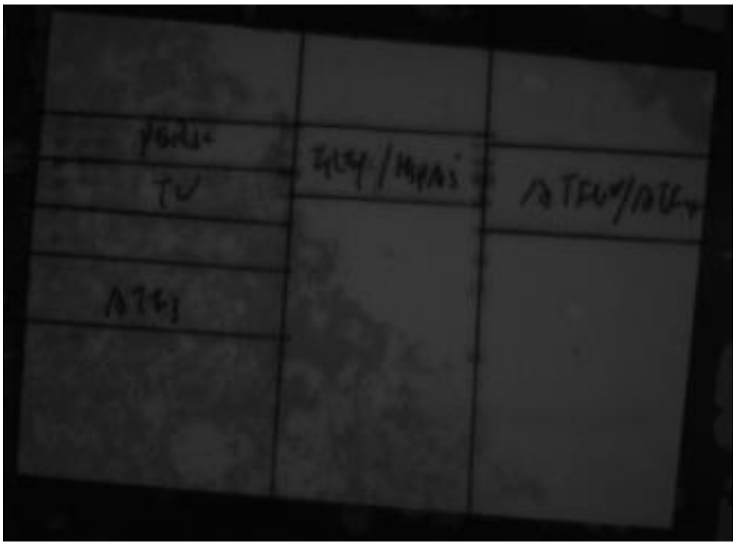

Figure 4D and 4E

β-Tubulin

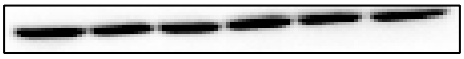

55KD

ATF3

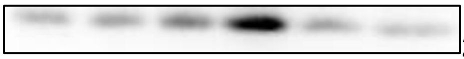

23,24KD

β-Tubulin

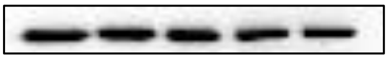

55KD

ATF3

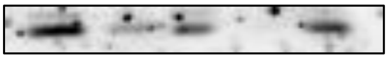

23,24KD

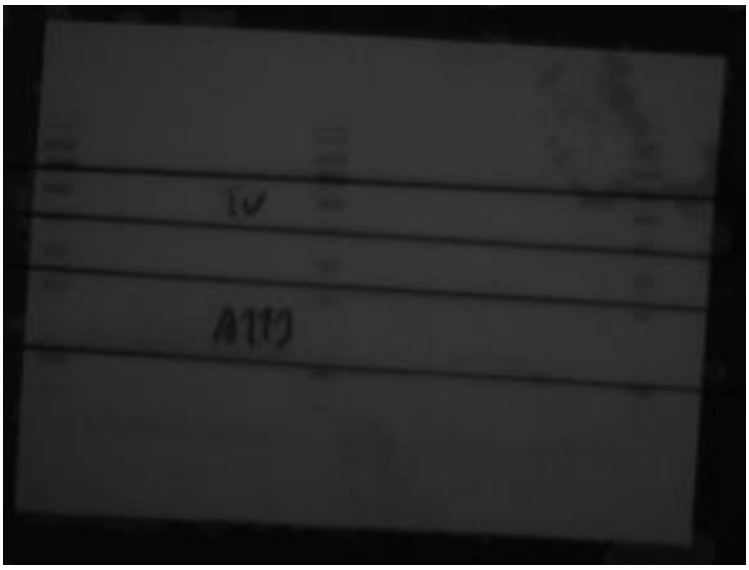

Figure 4H

β-Tubulin

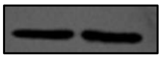

55KD

Flag

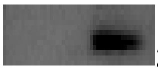

24,25KD

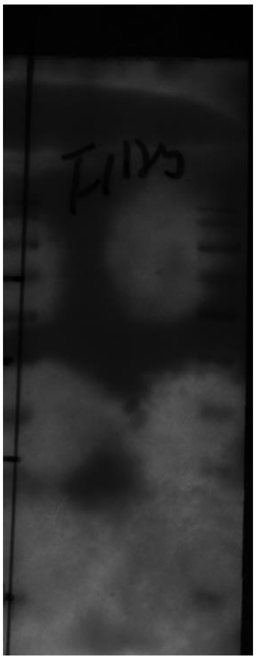

Figure 5E

β-Tubulin

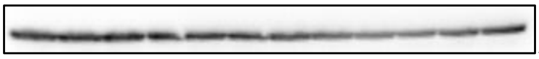

55KD

ATF3

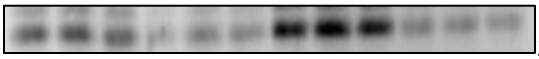

23,24KD

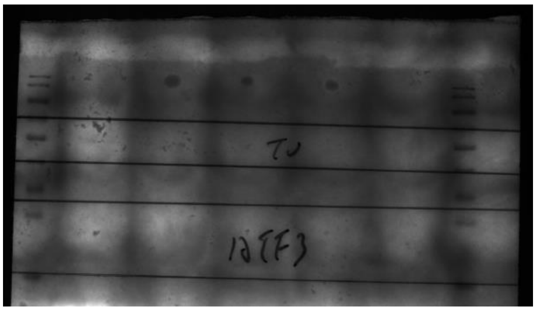

Supplement: Supplementary file 1 — Supplementary figures and table. [file jcav14p1336s1.pdf]
